# Neural processes linking joint hypermobility and anxiety: key roles for the amygdala and insular cortex

**DOI:** 10.1192/bjp.2024.259

**Published:** 2026-02

**Authors:** Christina N. Kampoureli, Charlotte L. Rae, Cassandra Gould Van Praag, Neil A. Harrison, Sarah N. Garfinkel, Hugo D. Critchley, Jessica A. Eccles

**Affiliations:** Department of Clinical Neuroscience, Brighton & Sussex Medical School, Falmer, UK; School of Psychology, University of Sussex, Falmer, UK; The Alan Turing Institute, British Library, London, UK; CUBRIC, University of Cardiff, UK; Institute of Cognitive Neuroscience, University College London, London, UK; Sussex Partnership NHS Foundation Trust, Worthing, UK

**Keywords:** Hypermobility, anxiety, EDS, fMRI, insula

## Abstract

**Background:**

Anxiety symptoms are elevated among people with joint hypermobility. The underlying neural mechanisms are attributed theoretically to effects of variant connective tissue on the precision of interoceptive representations contributing to emotions.

**Aim:**

To investigate the neural correlates of anxiety and hypermobility using functional neuroimaging.

**Method:**

We used functional magnetic resonance neuroimaging to quantify regional brain responses to emotional stimuli (facial expressions) in people with generalised anxiety disorder (GAD) (*N* = 30) and a non-anxious comparison group (*N* = 33). All participants were assessed for joint laxity and were classified (using Brighton Criteria) for the presence and absence of hypermobility syndrome (HMS: now considered hypermobility spectrum disorder).

**Results:**

Participants with HMS showed attenuated neural reactivity to emotional faces in specific frontal (inferior frontal gyrus, pre-supplementary motor area), midline (anterior mid and posterior cingulate cortices) and parietal (precuneus and supramarginal gyrus) regions. Notably, interaction between HMS and anxiety was expressed in reactivity of the left amygdala (a region implicated in threat processing) and mid insula (primary interoceptive cortex) where activity was amplified in people with HMS with GAD. Severity of hypermobility in anxious, compared with non-anxious, individuals correlated with activity within the anterior insula (implicated as the neural substrate linking anxious feelings to physiological state). Amygdala-precuneus functional connectivity was stronger in participants with HMS, compared with non-HMS participants.

**Conclusions:**

The predisposition to anxiety in people with variant connective tissue reflects dynamic interactions between neural centres processing threat (amygdala) and representing bodily state (insular and parietal cortices). Correspondingly, interventions to regulate amygdala reactivity while enhancing interoceptive precision may have therapeutic benefit for symptomatic hypermobile individuals.

Joint hypermobility is one visible manifestation of familial connective tissue variants that can impact organ function throughout the body. Joint hypermobility often results in troublesome joint pain and stiffness,^1^ yet remains under-recognised and poorly managed. The diagnosis of joint hypermobility syndrome (HMS), as defined by the Brighton criteria, requires joint hypermobility plus musculoskeletal or connective tissue symptoms (e.g. prolapse, easy bruising, dislocations).^2^ This classification has now been superseded by the term Hypermobility Spectrum Disorder.^3^ Rates of anxiety are markedly higher (odds ratio of 4.39) among hypermobile individuals.^4^ There is an overrepresentation of individuals with hypermobility in people with anxiety-related conditions and in presentations in which anxiety frequently co-occurs, including neurodevelopmental conditions such as autism and attention-deficit hyperactivity disorder.^5,6^ Furthermore, individuals with hypermobility often experience symptoms of dysautonomia, such as postural tachycardia syndrome.^7^ One explanation, connecting joint hypermobility, autonomic dysfunction and anxiety, proposes that relative inelasticity of connective tissue within peripheral vasculature compromises vasoconstriction and reduces venous return during standing through venous pooling. Compensatory autonomic responses, including increased sympathetic activity, increases physiological arousal including heart rate.^8–11^

In addition, hypermobile individuals may also show differences in interoceptive attention and sensitivity (increased sensing of changes from within the body^12^), reflecting the experience of greater interoceptive surprise through less predictable (more imprecise) afferent visceral signals.^13^ Increased attention may amplify interoceptive prediction error signals that contribute to the feeling of anxiety. Correspondingly, within the brain, hypermobile individuals are reported to show heightened reactivity in response to affective stimulation, both in regions responsible for interoceptive representation (insular cortex) and for emotional processing (amygdala),^14^ where structural differences are reported even in people with subclinical hypermobile features.^15^

The aim of this study is to use functional neuroimaging to explore the neural basis for the relationship between joint hypermobility and clinical anxiety, building on this earlier work.^14,15^ We hypothesised first that participants with anxiety would exhibit heightened insula and amygdala reactivity when processing social emotional stimuli (facial expressions), replicating prior findings.^13,16–18^ No previous functional imaging work has specifically addressed the link between hypermobility and clinical anxiety; we therefore additionally hypothesised that the reactivity of the amygdala and insula in conjunction with engagement of other ‘body-related’ brain regions would vary according to the presence and absence of hypermobility and anxiety, thereby illuminating neural substrates underlying their interaction.^14,15^

## Method

### Participants

Sixty-three participants were recruited to the study. People volunteered to participate in response to an advertisement from Sussex Partnership NHS Trust either after inclusion in a linked study or via electronic bulletin boards. Members of the non-clinical comparison group were recruited via electronic bulletin boards. Of the 63 participants, 30 (47.6%) participants (age; mean ± s.e.m.) = 42.93 ± 2.24 yrs, 18 female, 12 male) met the threshold for generalised anxiety disorder (GAD), and 33 (52.4%) participants (age; mean ± s.e.m. = 37.42 ± 2.28 yrs, 16 female, 17 male) were healthy controls. There were no statistically significant differences in age or gender between the two groups. Of the people with GAD, 18 (60%) were classified as having joint hypermobility syndrome, and 7 (21.2%) of the non-clinical comparison group met the diagnostic threshold for joint hypermobility syndrome. See Table 1 for participant demographic details and clinical features.

**Table 1:** Demographic details and anxiety level (measured using the Beck Anxiety Inventory (BAI)) of participants with generalised anxiety disorder (GAD) and the non-clinical comparison group. Group difference *P*-values refer to a two-tailed *t*-test (age) or *χ*^2^ test (gender)

Group	GAD: HMS	GAD: non-HMS	Comparison: HMS	Comparison: non-HMS	Group difference
Number	18	12	7	26	
	30		33		
Age (mean, s.e.m.)	43 ± 2		37 ± 2		*t*(61) = −1.72, *P* = 0.091
Gender	18 female; 12 male		16 female; 17 male		*χ*^2^(1) = 0.84, *P* = 0.360
BAI (mean, s.e.m.)	22.9 (1.78)		5.48 (0.70)		*t*(61) = −9.11, *P* < 0.001

HMS, hypermobility syndrome.

Inclusion criteria for people included the DSM-IV diagnosis of GAD. Participants in the non-clinical comparison group were required to be free from any history of psychiatric disorder. General exclusion criteria included magnetic resonance imaging (MRI) incompatibility, presence of neurological illness and presence of diagnosed psychiatric illness other than anxiety or comorbid depression in people. Written informed consent was obtained from all participants. The authors assert that all procedures contributing to this work comply with the ethical standards of the relevant national and institutional committees on human experimentation and with the Helsinki Declaration of 1975, as revised in 2013. All procedures involving human participants including individuals were approved by the National Research Ethics Service – South East Coast (Brighton and Sussex; REC reference 12/LO/1942; IRAS registration number 115219).

All participants underwent assessment for GAD and hypermobility. DSM-IV diagnosis of GAD was confirmed or refuted using the Mini International Neuropsychiatric Interview (MINI).^19^ The presence or absence of generalised joint laxity was established through physical examination of joints using the Beighton Scale ^20^ where a cut-off of 4 out of 9 was used in line with the UK literature, e.g. Clinch et al.^21^ All participants were assessed by the same clinician (J.A.E.). The presence of HMS was confirmed or excluded using Brighton Criteria.^2^

Anxiety level was measured using the Beck Anxiety Inventory (BAI).^22^There was significant group difference in anxiety levels (BAI; mean ± s.e.m.: anxious group 22.9 ± 1.78 versus non-anxious group 5.48 ± 0.70; *t*(61) = 9.11, *P* < 0.001). See Table 1 for anxiety levels measured using the BAI across groups. There was no significant difference in anxiety levels between those with HMS and those without, either in the anxious (mean ± s.e.m.: anxious group: HMS 23.28 ± 2.37 versus anxious group: non-HMS 22.42 ± 2.82; *t*(28) = −0.23, *P* = 0.818) or non-anxious group (non-anxious group: HMS 5.38 ± 0.74 versus non-anxious group: non-HMS 5.86 ± 1.92; *t*(31) = −0.27, *P* = 0.786). All clinical and demographic data were analysed with IBM SPSS Statistics v. 29.^23^

### Emotional faces task

An emotional faces task was modified from Umeda and colleagues,^24^ wherein five classes of images of emotional faces from the Karolinska Directed Emotional Faces set (KDEF) (classes: angry, afraid, disgusted, happy and neutral)^25^ were presented in a randomised order. Null events were pseudo-randomised and presented as fixation cross. These were also included to facilitate the identification of haemodynamic responses to stochastically ordered stimuli. There were 15 trials of each emotion category, and 21 null events, each lasting 4 s. During each face presentation, participants were asked to make an incidental judgement of whether they could see teeth or not (index or middle finger button press with right hand) to ensure attention to the stimuli**.**

### MRI acquisition

Functional MRI data were acquired on a Siemens Avanto 1.5 Tesla with a 32-channel head coil (T_2_*-weighted echo planar images, repetition time 2520 ms, echo time 43 ms, 34 interleaved slices 3 mm thick with 0.6 mm interslice gap, in-plane resolution 3 × 3 mm). A T_1_ structural was acquired for registration (repetition time 2730 ms, echo time 3.57 ms, 1 × 1 × 1 mm resolution).

### fMRI preprocessing

Functional MRI data were preprocessed and analysed using Statistical Parametric Mapping (SPM12)^26^ running in MATLAB.^27^ Preprocessing was performed using default options, including realignment to the mean image, slice-time correction to the 6th slice, co-registration to the T1 structural image and normalisation to Montreal Neurological Institute space, as well as smoothing at 8 mm Gaussian smoothing kernel. To account for head motion, framewise displacement (FD) values were calculated from the six motion parameters (rp_.txt) generated in SPM during realignment using the FD_conn method in the CONN functional connectivity toolbox running in SPM12 (version 22.a).^28^ Participants with excessive motion (framewise displacement > 0.5 mm) were flagged for closer inspection. Two participants exceeded this threshold (with framewise displacement values of 0.63 mm and 0.60 mm respectively) but were retained in the analysis to preserve the representativeness and generalisability of the findings within the clinical population.

### Statistical analysis

#### First-level general linear model

Task events were modelled in a general linear model, with five regressors representing the onset and duration of presentation of angry, afraid, disgusted, neutral and happy faces respectively. To account for head motion, six nuisance regressors modelled head movement using the motion parameters calculated during realignment. Single-regressor T-contrasts were generated for viewing (a) angry, (b) afraid, (c) disgusted, (d) neutral and (e) happy faces by assigning a contrast weight of 1 to each of the five experimental conditions, with the intertrial interval fixation cross representing an implicit baseline. These T-contrasts were entered into a full factorial second-level analysis.

#### Second-level general linear model

A second-level full factorial model contained HMS (non-HMS, HMS) and anxiety (non-anxious, anxious) as an independent (between-subjects) factor, and facial expression (angry, afraid, disgusted, neutral and happy) as a within-subject factor. In addition, two covariates were entered for (a) gender (male, female) and (b) age.

F-contrasts were generated testing for: all effects, main effect of HMS, main effect of anxiety, main effect of task and interactions between the factors. Individual group effects for viewing faces (compared with implicit baseline) were examined using T-contrasts: HMS > non-HMS; HMS (anxious > non-anxious); non-HMS > HMS; and anxious (HMS > non-HMS). A series of two further second-level models included the Beighton score and anxiety level (BAI score) as additional covariates. In the Beighton second-level model, the Beighton score was used as a covariate so that the main effect of hypermobility symptoms could be modelled along with the interaction of hypermobility symptoms with the anxiety factor (i.e. presence of GAD or not). In the anxiety second-level model, the anxiety level (BAI score) was used as a covariate, so that the main effect of the anxiety level could be modelled along with the interaction of the anxiety level (BAI score) with the factor of HMS. All covariates were mean centred around zero.

Statistical images were thresholded at a cluster-forming threshold of *P* < 0.001 for family-wise error rate correction (FWEc) for multiple comparisons at *P* < 0.05.^29^ Significant clusters were localised according to the Anatomy toolbox running in SPM12 (v 3.0).^30^

### Psychophysiological interactions

We performed a series of psychophysiological interaction analyses to investigate how brain activity in response to emotional faces, within regions identified in the above univariate analyses, changed in their functional connectivity to other regions of the brain as a function of hypermobility and anxiety status. On the basis of the univariate fMRI results, we identified three regions from which to seed these functional connectivity analyses: (a) left amygdala (centred on *x* −32, *y* 0, *z* −16); (b) right mid insula (centred on *x* 34, *y* −2, *z* −6); and (c) left inferior frontal gyrus (*x* −46, *y* 34, *z* 2). First, we extracted the first eigenvariate (weighted mean of blood-oxygen-level-dependent (BOLD) time series) for each region by thresholding three contrasts at *P* < 1 for each participant: (a) the interaction between anxiety and HMS (for left amygdala region of interest (ROI)); (b) the main effect of HMS for (right mid insula ROI); and (c) the main effect of HMS (for left inferior frontal gyrus ROI). Then, an F-contrast was computed for each subject, representing all effects (angry, afraid, disgusted, neutral and happy: ‘eye:5’). In the three contrasts given above, we then extracted a 10 mm sphere of voxels for each ROI, adjusting for the F-contrast of all effects.

Next, the psychophysiological interaction term was calculated according to the main effect of the task (contrast weights: 1 for angry, 1 for afraid, 1 for disgusted, 1 for neutral and 1 for happy) and the BOLD time series for each ROI. These psychophysiological interaction terms were each entered into a first-level model for each participant, alongside a regressor representing the BOLD activity of the ROI (psychophysiological interation, original region of interest eigenvariate (PPI.Y)) and the main effect of the task (psychophysiological interation, attention–no attention task vector (PPI.P)). Single regressor T-contrasts were generated for the psychophysiological interaction term using a single contrast weight to investigate positive changes in the regression slope of voxels elsewhere in the brain relative to the seed ROI during task events relative to baseline.

The first-level T-contrasts were then entered into a series of second-level models that examined the psychophysiological interaction between the seed ROI and voxels across the brain using a full factorial second-level analysis, with HMS (non-HMS, HMS) and anxiety (non-anxious, anxious) as an independent (between-participant) factor, and the first-level T-contrasts representing functional connectivity when viewing faces as a repeated measures (within-participant) factor.

In these second-level models, as with the univariate functional MRI analysis, age and gender were entered as covariates. Contrasts were thresholded at a cluster-forming threshold of *P* < 0.001 for FWEc at *P* < 0.05. Significant clusters were localised with reference to the SPM Anatomy toolbox (v 2.2b).^30^

#### Transparency declaration

We affirm that the manuscript is an honest, accurate and transparent account of the study being reported, and that no important aspects of the study have been omitted.

## Results

### Univariate functional MRI

#### Main effects

As anticipated, there was a significant main effect of anxiety within the left amygdala and right mid insula, with *post hoc* T-contrasts revealing greater activity in these regions in anxious versus non-anxious participants (Supplementary Material, Tables 4 and 5 available at https://doi.org/10.1192/bjp.2024.259).

**Fig. 1:**
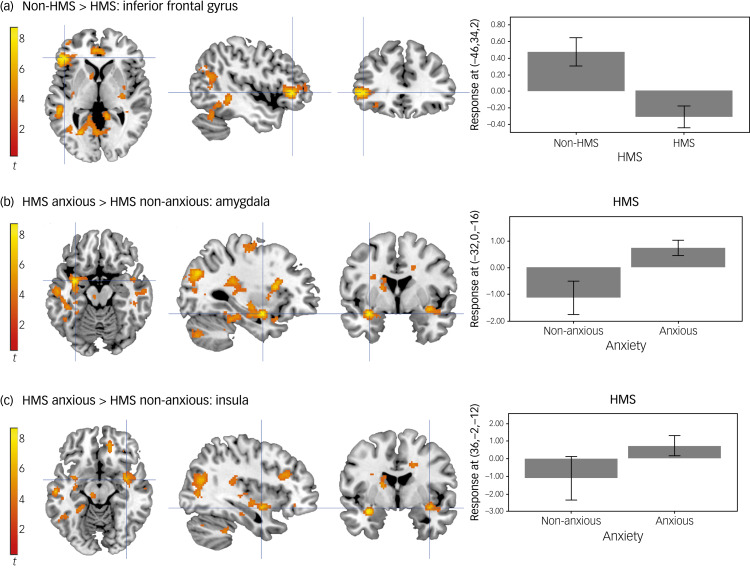
Activity while viewing emotional faces. (a) Activation in the inferior frontal gyrus is greater in non-HMS participants compared with the HMS. (b) Activation in the amygdala is greater in the HMS anxious participants compared with the HMS non-anxious participants. (c) Activation in the mid-insula is greater in the HMS anxious participants compared with the HMS non-anxious participants. Unthresholded statistic images are openly available at https://identifiers.org/neurovault.collection:16863. HMS refers to the diagnosis of joint hypermobility syndrome.

We also observed significant main effects of HMS in the left inferior frontal gyrus, precuneus and pre-supplementary motor area (SMA), right mid insula, right posterior and left anterior mid cingulate gyrus and the left supramarginal gyrus (Supplementary Material, Table 1). *Post hoc* T-contrasts revealed that non-HMS participants showed greater activity in the left inferior frontal gyrus, precuneus, left pre-SMA, right posterior and left anterior mid cingulate gyrus and the left supramarginal gyrus when viewing emotional faces compared with participants with HMS (Fig. 1(a)). *Post hoc* T-contrasts for the right mid insula were not statistically significant.

We also observed the known main effect of viewing emotional faces, associated with activation of large areas of the occipital lobe, right middle and left superior frontal gyrus (Supplementary Material, Table 3).

#### Interaction: hypermobility × anxiety

Furthermore, there was a significant interaction between HMS and anxiety in the left amygdala, left hippocampus, right paracingulate gyrus and right mid insula (Supplementary Material, Table 6). *Post hoc* T-contrasts revealed that: (a) in the group with HMS, there was greater activation in the left amygdala and the right paracingulate gyrus in anxious compared with non-anxious participants (Fig. 1(b)); (b) findings for the left hippocampus were not significant; (c) in participants with anxiety, there was greater activation in the right paracingulate gyrus and right mid-insula in the group with HMS compared with the non-HMS group (Fig. 1(c)).

#### Interaction: Beighton score × anxiety

**Fig. 2:**
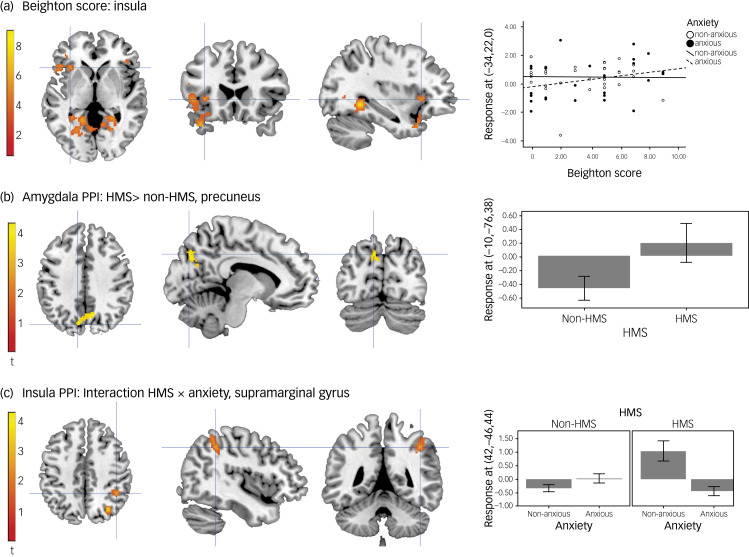
(a) Activation in anterior insula: interaction between Beighton score and anxiety status. (b) Changes in functional connectivity between the amygdala and the precuneus in HMS participants compared with non-HMS. (c) Changes in functional connectivity between the insula and supramarginal gyrus and the interaction between HMS and anxiety. Unthresholded statistic images are openly available at https://identifiers.org/neurovault.collection:16863. HMS refers to the diagnosis of joint hypermobility syndrome; PPI, psychophysiological interaction.

The interaction between the number of hypermobile joints (Beighton score) and anxiety status, i.e. the interaction testing for regions in which activation was more positively correlated with the Beighton score for anxious compared with non-anxious participants, showed activation in the left anterior insula (Fig. 2(a)).

#### Interaction: Beck anxiety score × hypermobility

The interaction between anxiety severity (BAI score) and HMS, i.e. the interaction testing for regions in which activation was more positively correlated with the anxiety score for people with HMS compared with non-HMS participants, showed activation in the left putamen (Supplementary Material, Table 10).

### Psychophysiological interactions

Three second-level models examined changes in functional connectivity with the (a) left amygdala, (b) right mid insula and (c) left inferior frontal gyrus, depending on the psychological context of viewing emotional faces. In the left amygdala psychophysiological interaction, there was no significant effect of HMS (F-contrast; HMS, non-HMS). However, the *post hoc* T-contrast testing for HMS versus non-HMS (T-contrast; HMS > non-HMS [−1 −1 1 1]) revealed that participants with HMS, when viewing faces, showed greater functional connectivity between the left amygdala and the left precuneus (Fig. 2(b)).

In the right mid insula psychophysiological interaction, there was a significant interaction effect between HMS and anxiety severity in the right supramarginal gyrus (Fig. 2(c)) and the right occipital cortex. However, the *post hoc* T-contrasts to explore the effect did not remain significant after correction for multiple comparisons. The left inferior frontal gyrus psychophysiological interaction did not produce statistically significant results.

## Discussion

Here, using functional neuroimaging during the incidental processing of emotional faces, we identify putative neural substrates underpinning the association between hypermobility and anxiety. Hypermobile participants showed reduced activation in discrete areas of the association cortex, notably the prefrontal and parietal regions. Anxious participants, in line with several previous imaging studies, showed amplified reactivity of the amygdala and insula during a socio-emotional challenge. For the first time, we were able to investigate the neural interaction between hypermobility and anxiety. We first confirmed that anxious participants with HMS showed greater activity in the amygdala and insula than non-anxious participants with HMS. Furthermore, the degree of hypermobility (as measured by the Beighton score) was more strongly correlated with insula activity in anxious participants with HMS than non-anxious participants with HMS. Finally, there was a general effect of hypermobility (regardless of anxiety) on functional connectivity between the amygdala and precuneus. Collectively, these findings identify a network of amygdala, insula and association cortices that link anxiety and hypermobility. Greater connectivity and activity within this network may underpin the increased prevalence of anxiety in individuals with HMS.

### Interoceptive pathways in the brain

The brain continuously receives sensory information from the visceral organs and peripheral tissues via ascending nerves that enter the brainstem.^31^ From here, interoceptive signals relaying the state of the body are conveyed to the thalamus, and ultimately the posterior insula cortex. Here, viscerotopic representations of these afferent signals are believed to support the cognitive perception of bodily feelings (e.g. heart rate, respiration, gastric sensations). This viscerotopic information is then re-represented, and integrated, more anteriorly in the insula lobe, underpinning our experience of broader affective states.^32,33^

Putatively, hypermobility may render individuals more prone to anxious affective experiences via heightened signalling of interoceptive signals relaying dysautonomic states. Because of changes in the connective tissue of the vasculature, hypermobile people may experience abnormal peripheral vasoconstriction.^34^ Specifically, reduced venous return during standing because of venous pooling may be responsible for an increased sympathetic state and autonomic hyperactivity.^8,9^ The insular cortex is an important central substrate for receiving this autonomic hyperactivity information.

It is therefore particularly intriguing that the insula was not only identified as overactive in our anxious sample, but more specifically, as more active in anxious versus non-anxious participants with HMS. We also saw that the degree of hypermobility (Beighton score) was more strongly correlated with insula activity in anxious than non-anxious participants with HMS. This identifies the insula as a nexus of affective experience in anxious hypermobile people. This finding extends similar observations reported in other affective conditions^35^ (e.g. increased emotional reactivity (hyperactivity of salience-processing regions) in bipolar disorder) and previous findings from our group that have examined the functional activity in non-anxious hypermobile participants.^14^ Within this context, interoception, i.e. the dynamic signalling, neural and perceptual representation of internal physiological states of the body, is a likely unifying factor. Additionally, participants with bipolar disorder have demonstrated abnormal insular functional connectivity, possibly modulated by inflammatory markers.^36^ Similar mechanisms may underpin the heightened insula activity observed in anxious hypermobile participants, potentially mediated by dysautonomic states.

### Amygdala interactions with hypermobility

In addition to reactivity differences in the insular cortex, we also observed interesting findings in the amygdala. Affective tasks performed during fMRI reliably engage the amygdala. This activation is typically amplified in anxious participants^37^ and is also enhanced following interoceptive stressors such as immune challenges.^38^ It is thus noteworthy that the amygdala was identified as a functional neural centre for interaction between anxiety and hypermobility, wherein the amygdala showed even greater activity in anxious participants with HMS than the non-anxious group with HMS. The amygdala is a critical region supporting the detection and perception of threat through associative integration of external and interoceptive information,^39^ which may underpin its role in affective experience.^40^ The greater reactivity of the amygdala in anxious hypermobile individuals may also reflect previously identified differences of amygdalar structure and function in hypermobile people.^14,15^ In parallel, the amygdala response in anxious participants with HMS may reflect the dynamic contributions (e.g. to behavioural/autonomic response and subjective feelings) of the amygdala and the insula within a wider affective network; however, our functional connectivity analyses did not identify a dependent association between these two regions.

Functional connectivity analyses did, however, identify stronger functional coupling between the amygdala and precuneus in hypermobile participants (regardless of anxiety). This may suggest that hypermobile individuals have a tendency towards hyper-connectivity of affective regions, which may have consequences for onward processing of information.

### Affective tasks as a probe for functional anatomy

We selected an emotional face processing task as a vehicle for probing affective responses in participants with GAD. A wide range of literature has previously taken such an approach to confirm the involvement of regions such as the amygdala and insula in a variety of anxiety conditions,^41^ as well as experimental interoceptive challenges.^38^ Here, we were able to extend this literature to understand the impact of hypermobility on these processes.

In our analyses, we collapsed across the five stimuli types to ask the fundamental question of how viewing social affective stimuli can provoke a neural response. In future work, it would be interesting to further tease apart the nuances of different affective cues on the hypermobile brain. For example, do responses to anger differ from fear?

### Limitations and future directions

Despite our implementation of a well-established paradigm to invoke reliable activations in affective brain regions, we acknowledge that fMRI tasks constrain one's investigative potential to the circumscribed set of regions that the task recruits. Other approaches, including resting-state fMRI, provide complementary information that can be leveraged to examine brain-wise network interactions. We did not acquire such data within this study, yet this remains an important avenue for future work to better understand the neural characteristics of hypermobility.

We recruited a community sample that enabled us to screen participants and place them into one of four categories according to anxiety and hypermobility status. However, since hypermobility is a risk factor for anxiety, with an odds ratio of 4.39 for suffering from anxiety if hypermobile,^4^ recruiting large numbers of hypermobile individuals who are not anxious (and anxious individuals who are not hypermobile) was a challenge. This means our sample sizes for the non-anxious HMS and anxious non-HMS groups were smaller than the other two groups.

Future work should capitalise on advances in biofeedback interventions (such as aligning dimensions of interoceptive experience^42^ and altering dynamics of autonomic processing therapies^43^) to target anxiety in hypermobile individuals, perhaps using neurofeedback to specifically down-regulate insular and amygdala reactivity. As in the broader mental health space, it is becoming increasingly clear that a ‘one-size-fits-all’ approach does not work for many groups experiencing anxiety; the present study will potentially inform personalised treatment approaches^44^ for a group of individuals who have previously perhaps been dismissed or overlooked.^45^

## Supporting information

Kampoureli et al. supplementary material Kampoureli et al. supplementary material

## Data Availability

The analytic code (SPM batches) that was used for the neuroimaging analysis for this study is available at OSF: osf.io/tcemx/. The MRI acquisition sequence information, demographic and clinical data, and participant mean framewise displacement values are available at OSF: osf.io/tcemx/. The neuroimaging data that support the findings of this study (unthresholded statistic images for every contrast reported) are openly available at https://identifiers.org/neurovault.collection:16863, reference number 16863.^46^

## References

[1] Keer R, Grahame R. Hypermobility Syndrome. Butterworth Heinemann, 2003.

[2] Grahame R, Bird HA, Child A. The revised (Brighton 1998) criteria for the diagnosis of benign joint hypermobility syndrome (BJHS). J Rheumatol 2000; 27(7): 1777–9. 10914867

[3] Castori M, Tinkle B, Levy H, Grahame R, Malfait F, Hakim A. A framework for the classification of joint hypermobility and related conditions. Am J Med Genet C Semin Med Genet 2017; 175(1): 148–57. 28145606 10.1002/ajmg.c.31539

[4] Smith TO, Easton V, Bacon H, Jerman E, Armon K, Poland F, et al. The relationship between benign joint hypermobility syndrome and psychological distress: a systematic review and meta-analysis. Rheumatology 2014; 53(1): 114–22. 24080253 10.1093/rheumatology/ket317

[5] Cederlöf M, Larsson H, Lichtenstein P, Almqvist C, Serlachius E, Ludvigsson JF. Nationwide population-based cohort study of psychiatric disorders in individuals with Ehlers–Danlos syndrome or hypermobility syndrome and their siblings. BMC Psychiatry 2016; 16(1): 207. 27377649 10.1186/s12888-016-0922-6 PMC4932739

[6] Csecs JLL, Iodice V, Rae CL, Brooke A, Simmons R, Quadt L, et al. Joint hypermobility links neurodivergence to dysautonomia and pain. Front Psychiatry 2022; 12: 786916. 35185636 10.3389/fpsyt.2021.786916 PMC8847158

[7] Hakim AJ. Non-musculoskeletal symptoms in joint hypermobility syndrome. Indirect evidence for autonomic dysfunction? Rheumatology 2004; 43(9): 1194–5. 15317957 10.1093/rheumatology/keh279

[8] Bohora S. Joint hypermobility syndrome and dysautonomia: expanding spectrum of disease presentation and manifestation. Indian Pacing Electrophysiol J 2010; 10(4): 158–61. 20376182 PMC2847865

[9] Mathias CJ, Low DA, Iodice V, Owens AP, Kirbis M, Grahame R. Postural tachycardia syndrome—current experience and concepts. Nat Rev Neurol 2012; 8(1): 22–34. 10.1038/nrneurol.2011.187 22143364

[10] Eccles JA, Owens AP, Mathias CJ, Umeda S, Critchley HD. Neurovisceral phenotypes in the expression of psychiatric symptoms. Front Neurosci 2015; 10(9): 4. 10.3389/fnins.2015.00004 PMC4322642 25713509

[11] Sharp HEC, Critchley HD, Eccles JA. Connecting brain and body: transdiagnostic relevance of connective tissue variants to neuropsychiatric symptom expression. World J Psychiatry 2021; 11(10): 805–20. 34733643 10.5498/wjp.v11.i10.805 PMC8546774

[12] Domschke K, Stevens S, Pfleiderer B, Gerlach AL. Interoceptive sensitivity in anxiety and anxiety disorders: an overview and integration of neurobiological findings. Clin Psychol Rev 2010; 30(1): 1–11. 19751958 10.1016/j.cpr.2009.08.008

[13] Paulus MP, Stein MB. An insular view of anxiety. Biol Psychiatry 2006; 60(4): 383–7. 16780813 10.1016/j.biopsych.2006.03.042

[14] Mallorquí-Bagué N, Garfinkel SN, Engels M, Eccles JA, Pailhez G, Bulbena A, et al. Neuroimaging and psychophysiological investigation of the link between anxiety, enhanced affective reactivity and interoception in people with joint hypermobility. Front Psychol 2014; 5: 1162. 25352818 10.3389/fpsyg.2014.01162 PMC4196473

[15] Eccles JA, Beacher FDC, Gray MA, Jones CL, Minati L, Harrison NA, et al. Brain structure and joint hypermobility: relevance to the expression of psychiatric symptoms. Br J Psychiatry 2012; 200(6): 508–9. 22539777 10.1192/bjp.bp.111.092460 PMC3365276

[16] Klumpp H, Post D, Angstadt M, Fitzgerald DA, Phan KL. Anterior cingulate cortex and insula response during indirect and direct processing of emotional faces in generalized social anxiety disorder. Biol Mood Anxiety Disord 2013; 3(1): 7. 23547713 10.1186/2045-5380-3-7 PMC3632493

[17] Shah SG, Klumpp H, Angstadt M, Nathan PJ, Phan KL. Amygdala and insula response to emotional images in patients with generalized social anxiety disorder. J Psychiatry Neurosci 2009; 34(4): 296–302. 19568481 PMC2702447

[18] Stein M. Increased amygdala and insula activation during emotion processing in anxiety-prone subjects. Am J Psychiatry 2007; 164(2): 318. 17267796 10.1176/ajp.2007.164.2.318

[19] Sheehan DV, Lecrubier Y, Sheehan KH, Amorim P, Janavs J, Weiller E, et al. The Mini-international neuropsychiatric interview (M.I.N.I.): the development and validation of a structured diagnostic psychiatric interview for DSM-IV and ICD-10. J Clin Psychiatry 1998; 59(Suppl 20): 22–33. quiz 34-57. 9881538

[20] Beighton P, Solomon L, Soskolne CL. Articular mobility in an African population. Ann Rheum Dis 1973; 32(5): 413–8. 4751776 10.1136/ard.32.5.413 PMC1006136

[21] Clinch J, Deere K, Sayers A, Palmer S, Riddoch C, Tobias JH, et al. Epidemiology of generalized joint laxity (hypermobility) in fourteen-year-old children from the UK: a population-based evaluation. Arthritis Rheum 2011; 63(9): 2819–27. 21547894 10.1002/art.30435 PMC3164233

[22] Beck AT, Epstein N, Brown G, Steer RA. An inventory for measuring clinical anxiety: psychometric properties. J Consult Clin Psychol 1988; 56(6): 893–7. 3204199 10.1037//0022-006x.56.6.893

[23] IBM Corporation. IBM SPSS Statistics. IBM, 2022.

[24] Umeda S, Harrison N, Gray M, Mathias C, Critchley H. Functional MRI investigations of emotional processing and autonomic responses in patients with autonomic hyperactivity. Neuroimage 2009; 47: S182.

[25] Goeleven E, De Raedt R, Leyman L, Verschuere B. The Karolinska directed emotional faces: a validation study. Cogn Emot 2008; 22(6): 1094–118.

[26] Penny W, Friston K, Ashburner J, Kiebel S, Nichols T. Statistical Parametric Mapping: The Analysis of Functional Brain Images. Academic Press, 2007.

[27] MATLAB. The MathWorks Inc, 2018.

[28] Whitfield-Gabrieli S, Nieto-Castanon A. Conn: a functional connectivity toolbox for correlated and anticorrelated brain networks. Brain Connect 2012; 2(3): 125–41. 22642651 10.1089/brain.2012.0073

[29] Eklund A, Nichols TE, Knutsson H. Cluster failure: why fMRI inferences for spatial extent have inflated false-positive rates. Proc Natl Acad Sci U S A 2016; 113(28): 7900–5. 27357684 10.1073/pnas.1602413113 PMC4948312

[30] Eickhoff SB, Paus T, Caspers S, Grosbras MH, Evans AC, Zilles K, et al. Assignment of functional activations to probabilistic cytoarchitectonic areas revisited. Neuroimage 2007; 36(3): 511–21. 17499520 10.1016/j.neuroimage.2007.03.060

[31] Craig AD. How do you feel? Interoception: the sense of the physiological condition of the body. Nat Rev Neurosci 2002; 3(8): 655–66. 12154366 10.1038/nrn894

[32] (Bud) Craig AD. How do you feel – now? The anterior insula and human awareness. Nat Rev Neurosci 2009; 10(1): 59–70. 19096369 10.1038/nrn2555

[33] Critchley HD, Garfinkel SN. Interoception and emotion. Curr Opin Psychol 2017; 17: 7–14. 28950976 10.1016/j.copsyc.2017.04.020

[34] Csecs JLL, Dowell NG, Savage GK, Iodice V, Mathias CJ, Critchley HD, et al. Variant connective tissue (joint hypermobility) and dysautonomia are associated with multimorbidity at the intersection between physical and psychological health. Am J Med Genet C Semin Med Genet 2021; 187(4): 500–9. 34806825 10.1002/ajmg.c.31957

[35] Förster K, Maliske LZ, Schurz M, Henneberg PM, Dannlowski U, Kanske P. How do bipolar disease states affect positive and negative emotion processing? Insights from a meta-analysis on the neural fingerprints of emotional processing. Bipolar Disord 2023; 25(7): 540–53. 37248623 10.1111/bdi.13341

[36] Chen P, Chen F, Chen G, Zhong S, Gong JY, Zhong H, et al. Inflammation is associated with decreased functional connectivity of insula in unmedicated bipolar disorder. Brain Behav Immun 2020; 89: 615–22. 32688026 10.1016/j.bbi.2020.07.004

[37] Fonzo GA, Etkin A. Affective neuroimaging in generalized anxiety disorder: an integrated review. Dialogues Clin Neurosci 2017; 19(2): 169–79. 28867941 10.31887/DCNS.2017.19.2/gfonzo PMC5573561

[38] Davies KA, Cooper E, Voon V, Tibble J, Cercignani M, Harrison NA. Interferon and anti-TNF therapies differentially modulate amygdala reactivity which predicts associated bidirectional changes in depressive symptoms. Mol Psychiatry 2021; 26: 5150–60. 32457424 10.1038/s41380-020-0790-9 PMC8589643

[39] Bach DR, Hurlemann R, Dolan RJ. Impaired threat prioritisation after selective bilateral amygdala lesions. Cortex 2015; 63: 206–13. 25282058 10.1016/j.cortex.2014.08.017 PMC4317193

[40] Garfinkel SN, Minati L, Gray MA, Seth AK, Dolan RJ, Critchley HD. Fear from the heart: sensitivity to fear stimuli depends on individual heartbeats. J Neurosc 2014; 34(19): 6573–82. 10.1523/JNEUROSCI.3507-13.2014 PMC4012313 24806682

[41] Engel K, Bandelow B, Gruber O, Wedekind D. Neuroimaging in anxiety disorders. J Neural Transm 2008; 116(6): 703–16. 18568288 10.1007/s00702-008-0077-9 PMC2694920

[42] Quadt L, Garfinkel SN, Mulcahy JS, Larsson DE, Silva M, Jones AM, et al. Interoceptive training to target anxiety in autistic adults (ADIE): a single-center, superiority randomized controlled trial-NC-ND license. EClinicalMedicine 2021; 39: 101042. 34401684 10.1016/j.eclinm.2021.101042 PMC8350004

[43] Davies G, Csecs JLL, Ball H, Dare J, Bremner S, Hosking R, et al. Altering dynamics of autonomic processing therapy (ADAPT) trial: a novel, targeted treatment for reducing anxiety in joint hypermobility. Trials 2021; 22(1): 645. 34548065 10.1186/s13063-021-05555-4 PMC8453027

[44] Williams LM, Carpenter WT, Carretta C, Papanastasiou E, Vaidyanathan U. Precision psychiatry and research domain criteria: implications for clinical trials and future practice. CNS Spectr 2023; 29(1): 26–39. 37675453 10.1017/S1092852923002420

[45] Halverson CME, Penwell HL, Francomano CA. Clinician-associated traumatization from difficult medical encounters: results from a qualitative interview study on the Ehlers-Danlos syndromes. SSM Qual Res Health 2023; 3: 100237. 37426705 10.1016/j.ssmqr.2023.100237 PMC10328215

[46] Hill SL, Laird AR, Marcus D, Gorgolewski KJ, Varoquaux G, Rivera G, et al. Neurovault.org: a web-based repository for collecting and sharing unthresholded statistical maps of the human brain. Front Neuroinf 2015; 9: 8. 10.3389/fninf.2015.00008 PMC4392315 25914639

